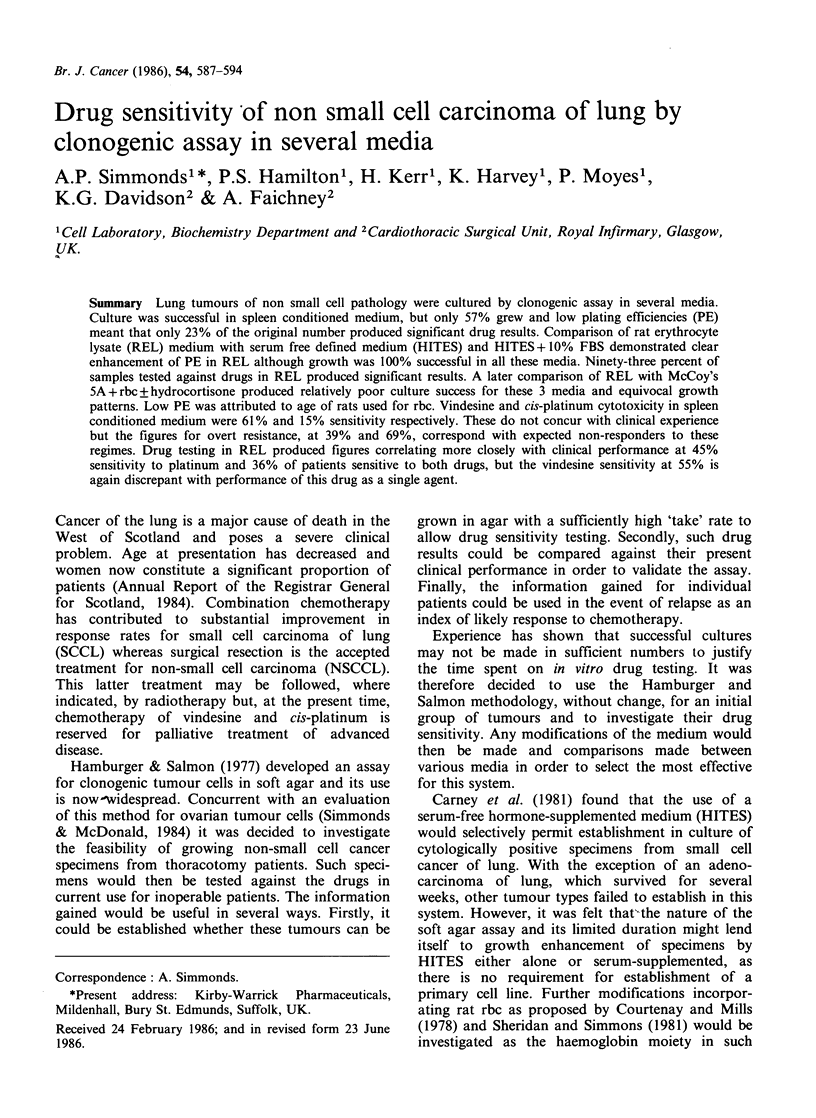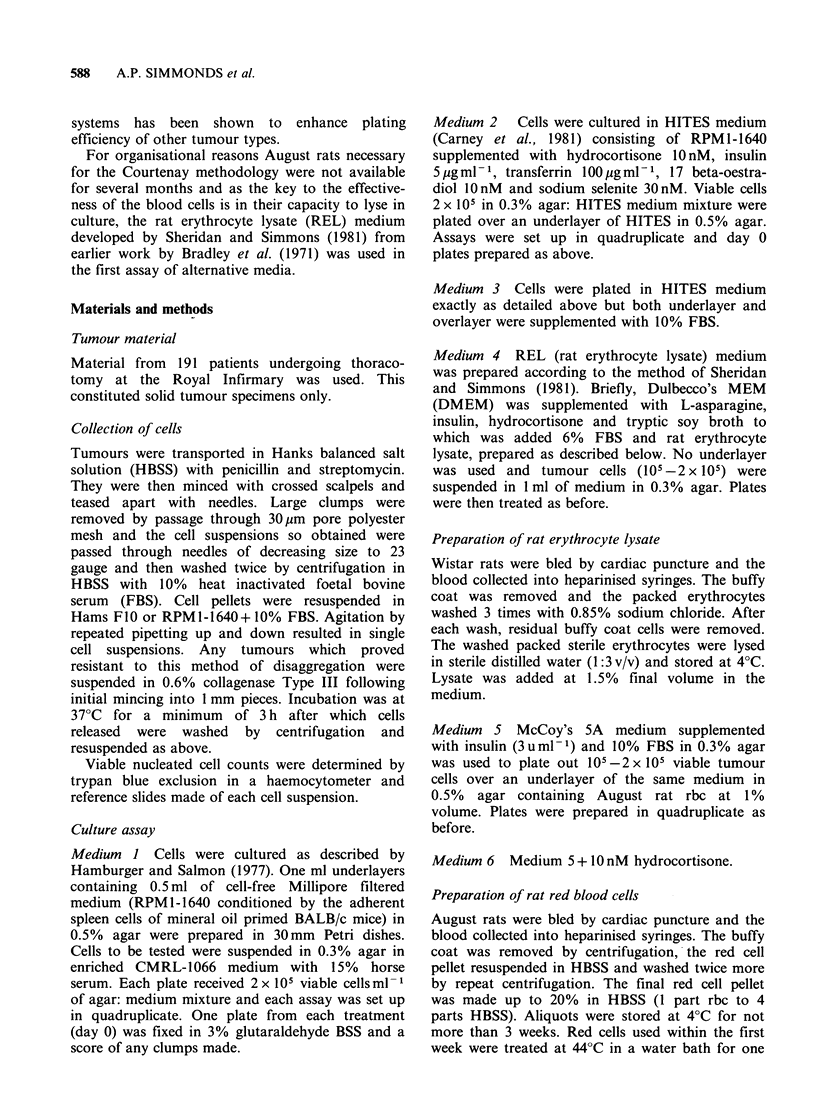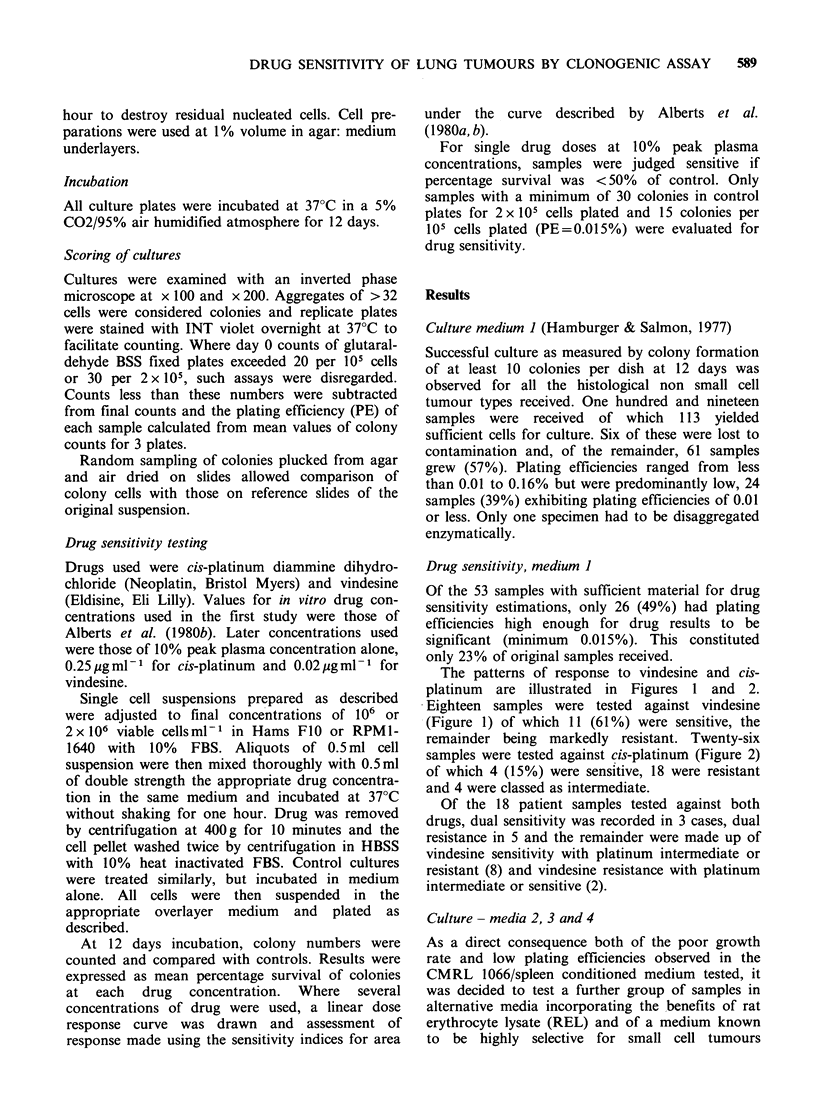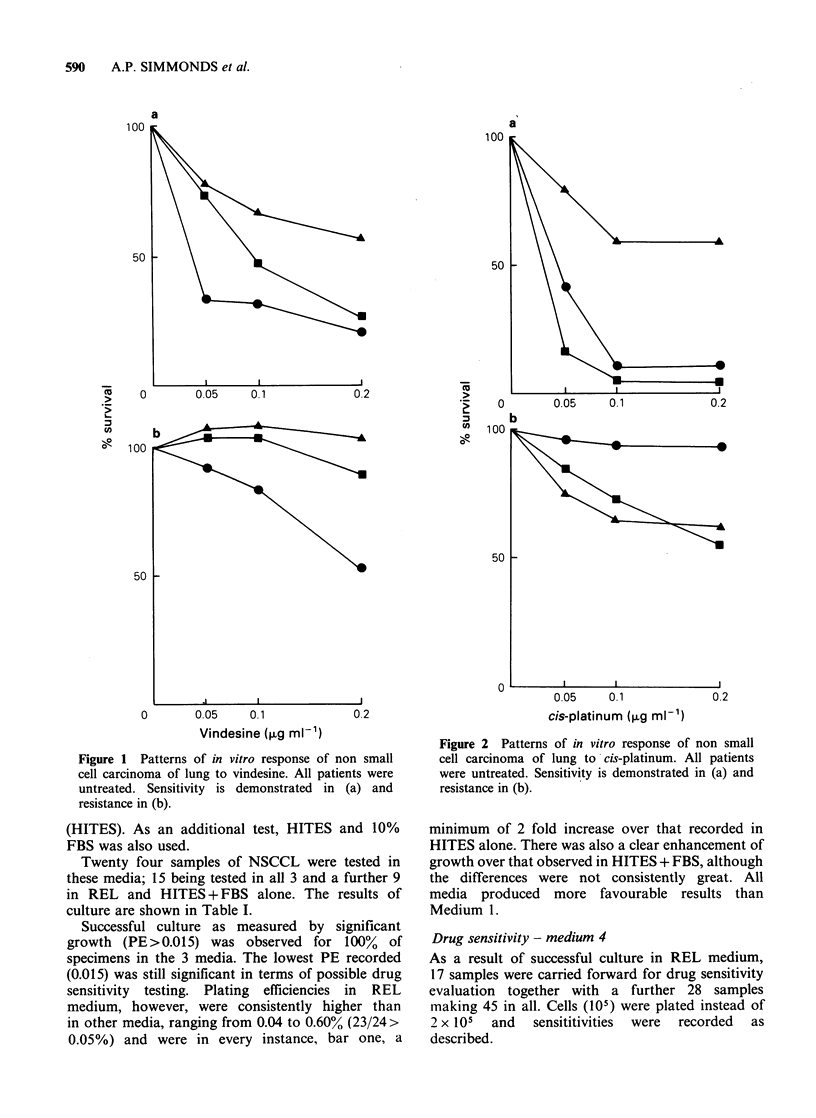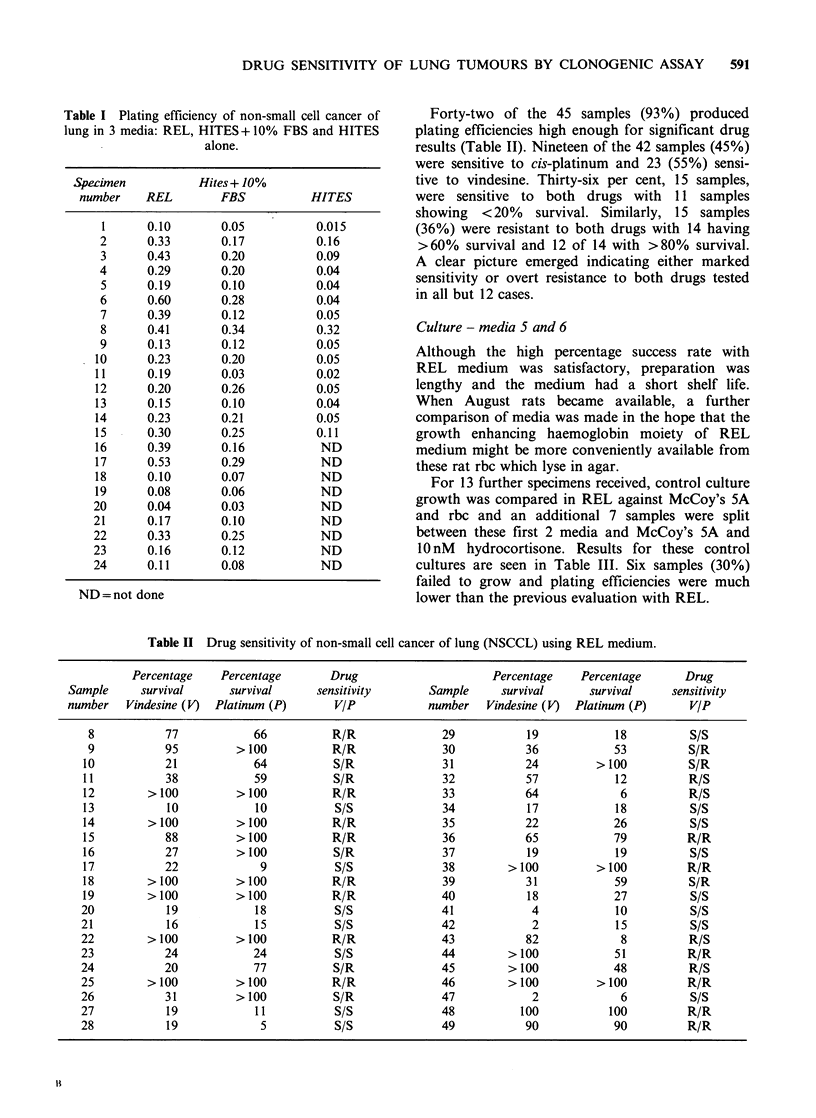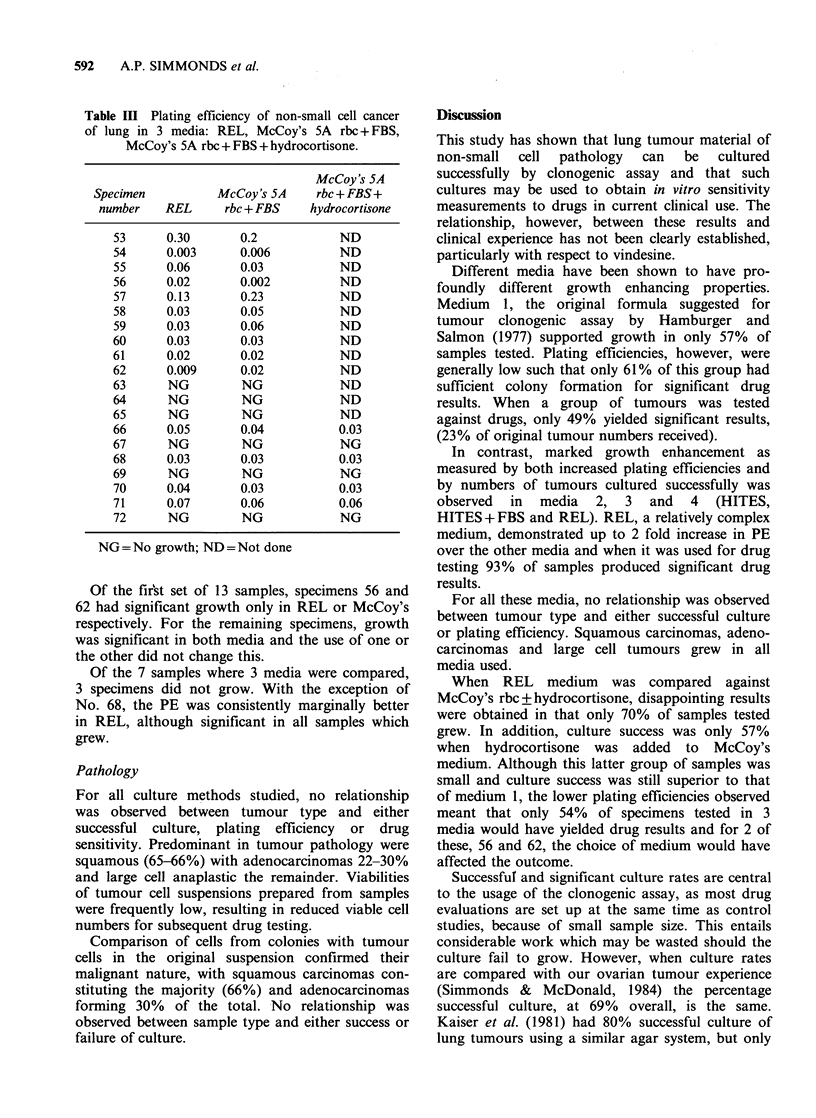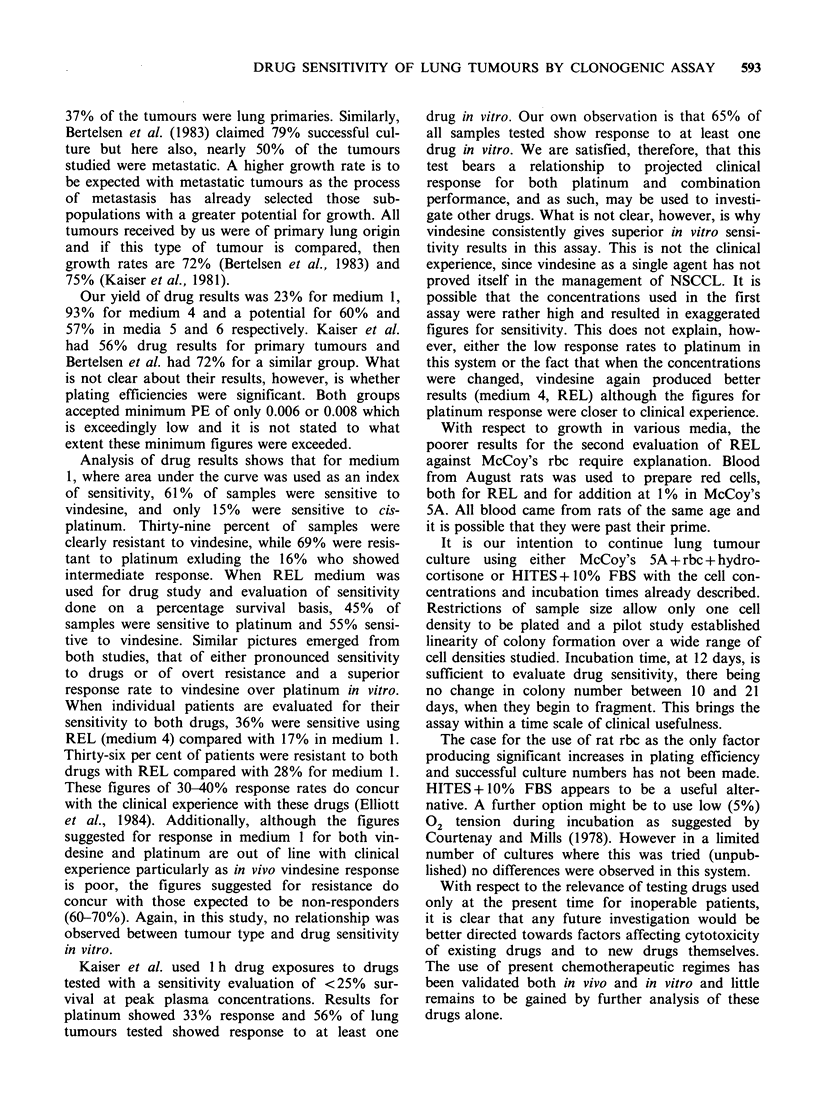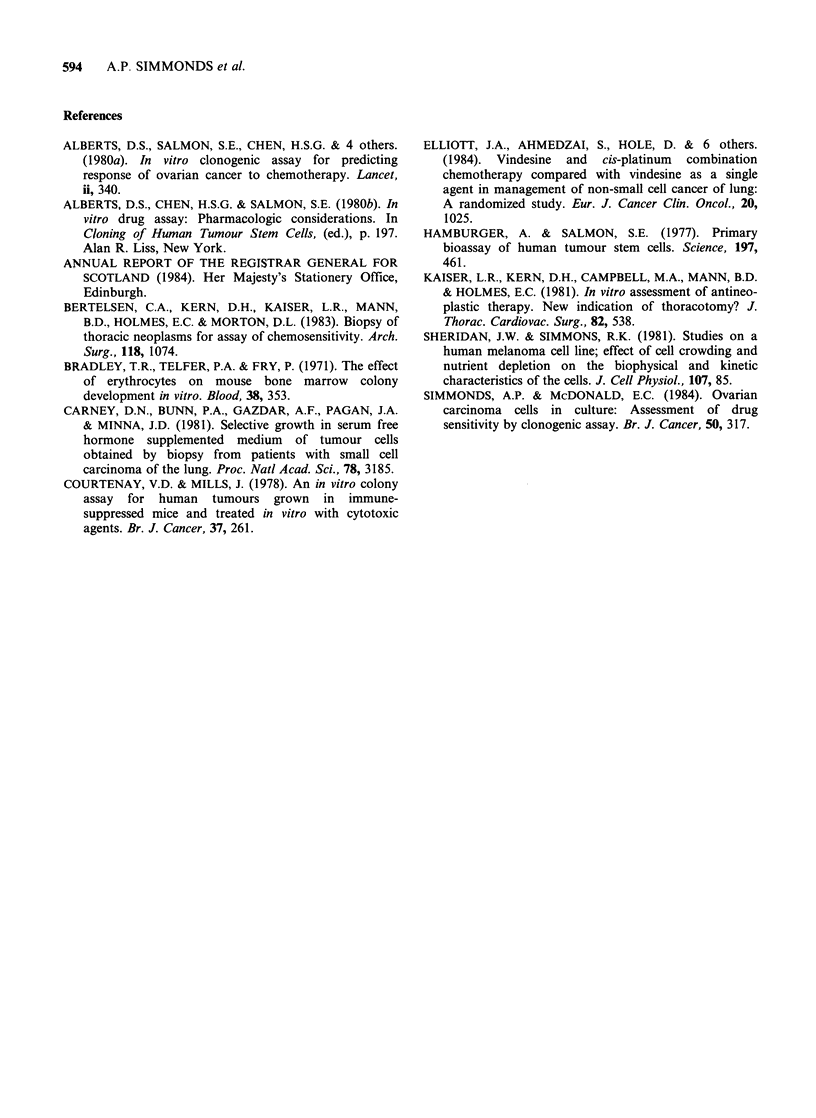# Drug sensitivity of non small cell carcinoma of lung by clonogenic assay in several media.

**DOI:** 10.1038/bjc.1986.213

**Published:** 1986-10

**Authors:** A. P. Simmonds, P. S. Hamilton, H. Kerr, K. Harvey, P. Moyes, K. G. Davidson, A. Faichney

## Abstract

Lung tumours of non small cell pathology were cultured by clonogenic assay in several media. Culture was successful in spleen conditioned medium, but only 57% grew and low plating efficiencies (PE) meant that only 23% of the original number produced significant drug results. Comparison of rat erythrocyte lysate (REL) medium with serum free defined medium (HITES) and HITES + 10% FBS demonstrated clear enhancement of PE in REL although growth was 100% successful in all these media. Ninety-three percent of samples tested against drugs in REL produced significant results. A later comparison of REL with McCoy's 5A + rbc +/- hydrocortisone produced relatively poor culture success for these 3 media and equivocal growth patterns. Low PE was attributed to age of rats used for rbc. Vindesine and cis-platinum cytotoxicity in spleen conditioned medium were 61% and 15% sensitivity respectively. These do not concur with clinical experience but the figures for overt resistance, at 39% and 69%, correspond with expected non-responders to these regimes. Drug testing in REL produced figures correlating more closely with clinical performance at 45% sensitivity to platinum and 36% of patients sensitive to both drugs, but the vindesine sensitivity at 55% is again discrepant with performance of this drug as a single agent.


Br. J. Cancer (1986), 54, 587–594

### Drug sensitivity of non small cell carcinoma of lung by clonogenic assay in several media

A.P. Simmonds[1]*, P.S. Hamilton[1], H. Kerr[1], K. Harvey[1], P. Moyes[1], K.G. Davidson[2] & A. Faichney[2]

[1]*Cell Laboratory, Biochemistry Department and* [2]*Cardiothoracic Surgical Unit, Royal Infirmary, Glasgow, UK.*

**Summary** Lung tumours of non small cell pathology were cultured by clonogenic assay in several media. Culture was successful in spleen conditioned medium, but only 57% grew and low plating efficiencies (PE) meant that only 23% of the original number produced significant drug results. Comparison of rat erythrocyte lysate (REL) medium with serum free defined medium (HITES) and HITES + 10% FBS demonstrated clear enhancement of PE in REL although growth was 100% successful in all these media. Ninety-three percent of samples tested against drugs in REL produced significant results. A later comparison of REL with McCoy's 5A + rbc ± hydrocortisone produced relatively poor culture success for these 3 media and equivocal growth patterns. Low PE was attributed to age of rats used for rbc. Vindesine and *cis*-platinum cytotoxicity in spleen conditioned medium were 61% and 15% sensitivity respectively. These do not concur with clinical experience but the figures for overt resistance, at 39% and 69%, correspond with expected non-responders to these regimes. Drug testing in REL produced figures correlating more closely with clinical performance at 45% sensitivity to platinum and 36% of patients sensitive to both drugs, but the vindesine sensitivity at 55% is again discrepant with performance of this drug as a single agent.

Cancer of the lung is a major cause of death in the West of Scotland and poses a severe clinical problem. Age at presentation has decreased and women now constitute a significant proportion of patients (Annual Report of the Registrar General for Scotland, 1984). Combination chemotherapy has contributed to substantial improvement in response rates for small cell carcinoma of lung (SCCL) whereas surgical resection is the accepted treatment for non-small cell carcinoma (NSCCL). This latter treatment may be followed, where indicated, by radiotherapy but, at the present time, chemotherapy of vindesine and *cis*-platinum is reserved for palliative treatment of advanced disease.

Hamburger & Salmon (1977) developed an assay for clonogenic tumour cells in soft agar and its use is now widespread. Concurrent with an evaluation of this method for ovarian tumour cells (Simmonds & McDonald, 1984) it was decided to investigate the feasibility of growing non-small cell cancer specimens from thoracotomy patients. Such specimens would then be tested against the drugs in current use for inoperable patients. The information gained would be useful in several ways. Firstly, it could be established whether these tumours can be grown in agar with a sufficiently high 'take' rate to allow drug sensitivity testing. Secondly, such drug results could be compared against their present clinical performance in order to validate the assay. Finally, the information gained for individual patients could be used in the event of relapse as an index of likely response to chemotherapy.

Experience has shown that successful cultures may not be made in sufficient numbers to justify the time spent on *in vitro* drug testing. It was therefore decided to use the Hamburger and Salmon methodology, without change, for an initial group of tumours and to investigate their drug sensitivity. Any modifications of the medium would then be made and comparisons made between various media in order to select the most effective for this system.

Carney *et al.* (1981) found that the use of a serum-free hormone-supplemented medium (HITES) would selectively permit establishment in culture of cytologically positive specimens from small cell cancer of lung. With the exception of an adenocarcinoma of lung, which survived for several weeks, other tumour types failed to establish in this system. However, it was felt that the nature of the soft agar assay and its limited duration might lend itself to growth enhancement of specimens by HITES either alone or serum-supplemented, as there is no requirement for establishment of a primary cell line. Further modifications incorporating rat rbc as proposed by Courtenay and Mills (1978) and Sheridan and Simmons (1981) would be investigated as the haemoglobin moiety in such

Correspondence: A. Simmonds.

*Present address: Kirby-Warrick Pharmaceuticals, Mildenhall, Bury St. Edmunds, Suffolk, UK.

Received 24 February 1986; and in revised form 23 June 1986.

systems has been shown to enhance plating efficiency of other tumour types.

For organisational reasons August rats necessary for the Courtenay methodology were not available for several months and as the key to the effectiveness of the blood cells is in their capacity to lyse in culture, the rat erythrocyte lysate (REL) medium developed by Sheridan and Simmons (1981) from earlier work by Bradley *et al.* (1971) was used in the first assay of alternative media.

### Materials and methods

### *Tumour material*

Material from 191 patients undergoing thoracotomy at the Royal Infirmary was used. This constituted solid tumour specimens only.

### *Collection of cells*

Tumours were transported in Hanks balanced salt solution (HBSS) with penicillin and streptomycin. They were then minced with crossed scalpels and teased apart with needles. Large clumps were removed by passage through 30 $\mu$m pore polyester mesh and the cell suspensions so obtained were passed through needles of decreasing size to 23 gauge and then washed twice by centrifugation in HBSS with 10% heat inactivated foetal bovine serum (FBS). Cell pellets were resuspended in Hams F10 or RPM1-1640 + 10% FBS. Agitation by repeated pipetting up and down resulted in single cell suspensions. Any tumours which proved resistant to this method of disaggregation were suspended in 0.6% collagenase Type III following initial mincing into 1 mm pieces. Incubation was at 37°C for a minimum of 3 h after which cells released were washed by centrifugation and resuspended as above.

Viable nucleated cell counts were determined by trypan blue exclusion in a haemocytometer and reference slides made of each cell suspension.

### *Culture assay*

*Medium 1* Cells were cultured as described by Hamburger and Salmon (1977). One ml underlayers containing 0.5 ml of cell-free Millipore filtered medium (RPM1-1640 conditioned by the adherent spleen cells of mineral oil primed BALB/c mice) in 0.5% agar were prepared in 30 mm Petri dishes. Cells to be tested were suspended in 0.3% agar in enriched CMRL-1066 medium with 15% horse serum. Each plate received $2 \times 10^5$ viable cells ml$^{-1}$ of agar: medium mixture and each assay was set up in quadruplicate. One plate from each treatment (day 0) was fixed in 3% glutaraldehyde BSS and a score of any clumps made.

*Medium 2* Cells were cultured in HITES medium (Carney *et al.*, 1981) consisting of RPM1-1640 supplemented with hydrocortisone 10 nM, insulin 5 $\mu$g ml$^{-1}$, transferrin 100 $\mu$g ml$^{-1}$, 17 beta-oestradiol 10 nM and sodium selenite 30 nM. Viable cells $2 \times 10^5$ in 0.3% agar: HITES medium mixture were plated over an underlayer of HITES in 0.5% agar. Assays were set up in quadruplicate and day 0 plates prepared as above.

*Medium 3* Cells were plated in HITES medium exactly as detailed above but both underlayer and overlayer were supplemented with 10% FBS.

*Medium 4* REL (rat erythrocyte lysate) medium was prepared according to the method of Sheridan and Simmons (1981). Briefly, Dulbecco's MEM (DMEM) was supplemented with L-asparagine, insulin, hydrocortisone and tryptic soy broth to which was added 6% FBS and rat erythrocyte lysate, prepared as described below. No underlayer was used and tumour cells ($10^5 - 2 \times 10^5$) were suspended in 1 ml of medium in 0.3% agar. Plates were then treated as before.

### *Preparation of rat erythrocyte lysate*

Wistar rats were bled by cardiac puncture and the blood collected into heparinised syringes. The buffy coat was removed and the packed erythrocytes washed 3 times with 0.85% sodium chloride. After each wash, residual buffy coat cells were removed. The washed packed sterile erythrocytes were lysed in sterile distilled water (1:3 v/v) and stored at 4°C. Lysate was added at 1.5% final volume in the medium.

*Medium 5* McCoy's 5A medium supplemented with insulin (3 u ml$^{-1}$) and 10% FBS in 0.3% agar was used to plate out $10^5 - 2 \times 10^5$ viable tumour cells over an underlayer of the same medium in 0.5% agar containing August rat rbc at 1% volume. Plates were prepared in quadruplicate as before.

*Medium 6* Medium 5 + 10 nM hydrocortisone.

### *Preparation of rat red blood cells*

August rats were bled by cardiac puncture and the blood collected into heparinised syringes. The buffy coat was removed by centrifugation, the red cell pellet resuspended in HBSS and washed twice more by repeat centrifugation. The final red cell pellet was made up to 20% in HBSS (1 part rbc to 4 parts HBSS). Aliquots were stored at 4°C for not more than 3 weeks. Red cells used within the first week were treated at 44°C in a water bath for one hour to destroy residual nucleated cells. Cell preparations were used at 1% volume in agar: medium underlayers.

*Incubation*

All culture plates were incubated at 37°C in a 5% CO2/95% air humidified atmosphere for 12 days.

*Scoring of cultures*

Cultures were examined with an inverted phase microscope at ×100 and ×200. Aggregates of >32 cells were considered colonies and replicate plates were stained with INT violet overnight at 37°C to facilitate counting. Where day 0 counts of glutaraldehyde BSS fixed plates exceeded 20 per $10^5$ cells or 30 per $2 \times 10^5$, such assays were disregarded. Counts less than these numbers were subtracted from final counts and the plating efficiency (PE) of each sample calculated from mean values of colony counts for 3 plates.

Random sampling of colonies plucked from agar and air dried on slides allowed comparison of colony cells with those on reference slides of the original suspension.

*Drug sensitivity testing*

Drugs used were *cis*-platinum diammine dihydrochloride (Neoplatin, Bristol Myers) and vindesine (Eldisine, Eli Lilly). Values for *in vitro* drug concentrations used in the first study were those of Alberts *et al.* (1980*b*). Later concentrations used were those of 10% peak plasma concentration alone, $0.25\,\mu g\,ml^{-1}$ for *cis*-platinum and $0.02\,\mu g\,ml^{-1}$ for vindesine.

Single cell suspensions prepared as described were adjusted to final concentrations of $10^6$ or $2 \times 10^6$ viable cells $ml^{-1}$ in Hams F10 or RPM1-1640 with 10% FBS. Aliquots of 0.5 ml cell suspension were then mixed thoroughly with 0.5 ml of double strength the appropriate drug concentration in the same medium and incubated at 37°C without shaking for one hour. Drug was removed by centrifugation at 400 g for 10 minutes and the cell pellet washed twice by centrifugation in HBSS with 10% heat inactivated FBS. Control cultures were treated similarly, but incubated in medium alone. All cells were then suspended in the appropriate overlayer medium and plated as described.

At 12 days incubation, colony numbers were counted and compared with controls. Results were expressed as mean percentage survival of colonies at each drug concentration. Where several concentrations of drug were used, a linear dose response curve was drawn and assessment of response made using the sensitivity indices for area under the curve described by Alberts *et al.* (1980*a, b*).

For single drug doses at 10% peak plasma concentrations, samples were judged sensitive if percentage survival was <50% of control. Only samples with a minimum of 30 colonies in control plates for $2 \times 10^5$ cells plated and 15 colonies per $10^5$ cells plated (PE=0.015%) were evaluated for drug sensitivity.

### Results

*Culture medium 1* (Hamburger & Salmon, 1977)

Successful culture as measured by colony formation of at least 10 colonies per dish at 12 days was observed for all the histological non small cell tumour types received. One hundred and nineteen samples were received of which 113 yielded sufficient cells for culture. Six of these were lost to contamination and, of the remainder, 61 samples grew (57%). Plating efficiencies ranged from less than 0.01 to 0.16% but were predominantly low, 24 samples (39%) exhibiting plating efficiencies of 0.01 or less. Only one specimen had to be disaggregated enzymatically.

*Drug sensitivity, medium 1*

Of the 53 samples with sufficient material for drug sensitivity estimations, only 26 (49%) had plating efficiencies high enough for drug results to be significant (minimum 0.015%). This constituted only 23% of original samples received.

The patterns of response to vindesine and *cis*-platinum are illustrated in Figures 1 and 2. Eighteen samples were tested against vindesine (Figure 1) of which 11 (61%) were sensitive, the remainder being markedly resistant. Twenty-six samples were tested against *cis*-platinum (Figure 2) of which 4 (15%) were sensitive, 18 were resistant and 4 were classed as intermediate.

Of the 18 patient samples tested against both drugs, dual sensitivity was recorded in 3 cases, dual resistance in 5 and the remainder were made up of vindesine sensitivity with platinum intermediate or resistant (8) and vindesine resistance with platinum intermediate or sensitive (2).

*Culture – media 2, 3 and 4*

As a direct consequence both of the poor growth rate and low plating efficiencies observed in the CMRL 1066/spleen conditioned medium tested, it was decided to test a further group of samples in alternative media incorporating the benefits of rat erythrocyte lysate (REL) and of a medium known to be highly selective for small cell tumours

**Figure 1** Patterns of *in vitro* response of non small cell carcinoma of lung to vindesine. All patients were untreated. Sensitivity is demonstrated in (a) and resistance in (b).

**Figure 2** Patterns of *in vitro* response of non small cell carcinoma of lung to *cis*-platinum. All patients were untreated. Sensitivity is demonstrated in (a) and resistance in (b).

(HITES). As an additional test, HITES and 10% FBS was also used.

Twenty four samples of NSCCL were tested in these media; 15 being tested in all 3 and a further 9 in REL and HITES+FBS alone. The results of culture are shown in Table I.

Successful culture as measured by significant growth (PE>0.015) was observed for 100% of specimens in the 3 media. The lowest PE recorded (0.015) was still significant in terms of possible drug sensitivity testing. Plating efficiencies in REL medium, however, were consistently higher than in other media, ranging from 0.04 to 0.60% (23/24> 0.05%) and were in every instance, bar one, a minimum of 2 fold increase over that recorded in HITES alone. There was also a clear enhancement of growth over that observed in HITES+FBS, although the differences were not consistently great. All media produced more favourable results than Medium 1.

*Drug sensitivity – medium 4*

As a result of successful culture in REL medium, 17 samples were carried forward for drug sensitivity evaluation together with a further 28 samples making 45 in all. Cells ($10^5$) were plated instead of $2 \times 10^5$ and sensititivities were recorded as described.

**Table I** Plating efficiency of non-small cell cancer of lung in 3 media: REL, HITES+10% FBS and HITES alone.

| *Specimen number* | *REL* | *Hites+10% FBS* | *HITES* |
|---|---|---|---|
| 1 | 0.10 | 0.05 | 0.015 |
| 2 | 0.33 | 0.17 | 0.16 |
| 3 | 0.43 | 0.20 | 0.09 |
| 4 | 0.29 | 0.20 | 0.04 |
| 5 | 0.19 | 0.10 | 0.04 |
| 6 | 0.60 | 0.28 | 0.04 |
| 7 | 0.39 | 0.12 | 0.05 |
| 8 | 0.41 | 0.34 | 0.32 |
| 9 | 0.13 | 0.12 | 0.05 |
| 10 | 0.23 | 0.20 | 0.05 |
| 11 | 0.19 | 0.03 | 0.02 |
| 12 | 0.20 | 0.26 | 0.05 |
| 13 | 0.15 | 0.10 | 0.04 |
| 14 | 0.23 | 0.21 | 0.05 |
| 15 | 0.30 | 0.25 | 0.11 |
| 16 | 0.39 | 0.16 | ND |
| 17 | 0.53 | 0.29 | ND |
| 18 | 0.10 | 0.07 | ND |
| 19 | 0.08 | 0.06 | ND |
| 20 | 0.04 | 0.03 | ND |
| 21 | 0.17 | 0.10 | ND |
| 22 | 0.33 | 0.25 | ND |
| 23 | 0.16 | 0.12 | ND |
| 24 | 0.11 | 0.08 | ND |

ND = not done

Forty-two of the 45 samples (93%) produced plating efficiencies high enough for significant drug results (Table II). Nineteen of the 42 samples (45%) were sensitive to *cis*-platinum and 23 (55%) sensitive to vindesine. Thirty-six per cent, 15 samples, were sensitive to both drugs with 11 samples showing <20% survival. Similarly, 15 samples (36%) were resistant to both drugs with 14 having >60% survival and 12 of 14 with >80% survival. A clear picture emerged indicating either marked sensitivity or overt resistance to both drugs tested in all but 12 cases.

*Culture – media 5 and 6*

Although the high percentage success rate with REL medium was satisfactory, preparation was lengthy and the medium had a short shelf life. When August rats became available, a further comparison of media was made in the hope that the growth enhancing haemoglobin moiety of REL medium might be more conveniently available from these rat rbc which lyse in agar.

For 13 further specimens received, control culture growth was compared in REL against McCoy's 5A and rbc and an additional 7 samples were split between these first 2 media and McCoy's 5A and 10 nM hydrocortisone. Results for these control cultures are seen in Table III. Six samples (30%) failed to grow and plating efficiencies were much lower than the previous evaluation with REL.

**Table II** Drug sensitivity of non-small cell cancer of lung (NSCCL) using REL medium.

| *Sample number* | *Percentage survival Vindesine (V)* | *Percentage survival Platinum (P)* | *Drug sensitivity V/P* | *Sample number* | *Percentage survival Vindesine (V)* | *Percentage survival Platinum (P)* | *Drug sensitivity V/P* |
|---|---|---|---|---|---|---|---|
| 8 | 77 | 66 | R/R | 29 | 19 | 18 | S/S |
| 9 | 95 | >100 | R/R | 30 | 36 | 53 | S/R |
| 10 | 21 | 64 | S/R | 31 | 24 | >100 | S/R |
| 11 | 38 | 59 | S/R | 32 | 57 | 12 | R/S |
| 12 | >100 | >100 | R/R | 33 | 64 | 6 | R/S |
| 13 | 10 | 10 | S/S | 34 | 17 | 18 | S/S |
| 14 | >100 | >100 | R/R | 35 | 22 | 26 | S/S |
| 15 | 88 | >100 | R/R | 36 | 65 | 79 | R/R |
| 16 | 27 | >100 | S/R | 37 | 19 | 19 | S/S |
| 17 | 22 | 9 | S/S | 38 | >100 | >100 | R/R |
| 18 | >100 | >100 | R/R | 39 | 31 | 59 | S/R |
| 19 | >100 | >100 | R/R | 40 | 18 | 27 | S/S |
| 20 | 19 | 18 | S/S | 41 | 4 | 10 | S/S |
| 21 | 16 | 15 | S/S | 42 | 2 | 15 | S/S |
| 22 | >100 | >100 | R/R | 43 | 82 | 8 | R/S |
| 23 | 24 | 24 | S/S | 44 | >100 | 51 | R/R |
| 24 | 20 | 77 | S/R | 45 | >100 | 48 | R/S |
| 25 | >100 | >100 | R/R | 46 | >100 | >100 | R/R |
| 26 | 31 | >100 | S/R | 47 | 2 | 6 | S/S |
| 27 | 19 | 11 | S/S | 48 | 100 | 100 | R/R |
| 28 | 19 | 5 | S/S | 49 | 90 | 90 | R/R |

**Table III** Plating efficiency of non-small cell cancer of lung in 3 media: REL, McCoy's 5A rbc+FBS, McCoy's 5A rbc+FBS+hydrocortisone.

| *Specimen number* | *REL* | *McCoy's 5A rbc+FBS* | *McCoy's 5A rbc+FBS+ hydrocortisone* |
|---|---|---|---|
| 53 | 0.30 | 0.2 | ND |
| 54 | 0.003 | 0.006 | ND |
| 55 | 0.06 | 0.03 | ND |
| 56 | 0.02 | 0.002 | ND |
| 57 | 0.13 | 0.23 | ND |
| 58 | 0.03 | 0.05 | ND |
| 59 | 0.03 | 0.06 | ND |
| 60 | 0.03 | 0.03 | ND |
| 61 | 0.02 | 0.02 | ND |
| 62 | 0.009 | 0.02 | ND |
| 63 | NG | NG | ND |
| 64 | NG | NG | ND |
| 65 | NG | NG | ND |
| 66 | 0.05 | 0.04 | 0.03 |
| 67 | NG | NG | NG |
| 68 | 0.03 | 0.03 | 0.03 |
| 69 | NG | NG | NG |
| 70 | 0.04 | 0.03 | 0.03 |
| 71 | 0.07 | 0.06 | 0.06 |
| 72 | NG | NG | NG |

NG = No growth; ND = Not done

Of the first set of 13 samples, specimens 56 and 62 had significant growth only in REL or McCoy's respectively. For the remaining specimens, growth was significant in both media and the use of one or the other did not change this.

Of the 7 samples where 3 media were compared, 3 specimens did not grow. With the exception of No. 68, the PE was consistently marginally better in REL, although significant in all samples which grew.

### *Pathology*

For all culture methods studied, no relationship was observed between tumour type and either successful culture, plating efficiency or drug sensitivity. Predominant in tumour pathology were squamous (65–66%) with adenocarcinomas 22–30% and large cell anaplastic the remainder. Viabilities of tumour cell suspensions prepared from samples were frequently low, resulting in reduced viable cell numbers for subsequent drug testing.

Comparison of cells from colonies with tumour cells in the original suspension confirmed their malignant nature, with squamous carcinomas constituting the majority (66%) and adenocarcinomas forming 30% of the total. No relationship was observed between sample type and either success or failure of culture.

### Discussion

This study has shown that lung tumour material of non-small cell pathology can be cultured successfully by clonogenic assay and that such cultures may be used to obtain *in vitro* sensitivity measurements to drugs in current clinical use. The relationship, however, between these results and clinical experience has not been clearly established, particularly with respect to vindesine.

Different media have been shown to have profoundly different growth enhancing properties. Medium 1, the original formula suggested for tumour clonogenic assay by Hamburger and Salmon (1977) supported growth in only 57% of samples tested. Plating efficiencies, however, were generally low such that only 61% of this group had sufficient colony formation for significant drug results. When a group of tumours was tested against drugs, only 49% yielded significant results, (23% of original tumour numbers received).

In contrast, marked growth enhancement as measured by both increased plating efficiencies and by numbers of tumours cultured successfully was observed in media 2, 3 and 4 (HITES, HITES+FBS and REL). REL, a relatively complex medium, demonstrated up to 2 fold increase in PE over the other media and when it was used for drug testing 93% of samples produced significant drug results.

For all these media, no relationship was observed between tumour type and either successful culture or plating efficiency. Squamous carcinomas, adenocarcinomas and large cell tumours grew in all media used.

When REL medium was compared against McCoy's rbc ± hydrocortisone, disappointing results were obtained in that only 70% of samples tested grew. In addition, culture success was only 57% when hydrocortisone was added to McCoy's medium. Although this latter group of samples was small and culture success was still superior to that of medium 1, the lower plating efficiencies observed meant that only 54% of specimens tested in 3 media would have yielded drug results and for 2 of these, 56 and 62, the choice of medium would have affected the outcome.

Successful and significant culture rates are central to the usage of the clonogenic assay, as most drug evaluations are set up at the same time as control studies, because of small sample size. This entails considerable work which may be wasted should the culture fail to grow. However, when culture rates are compared with our ovarian tumour experience (Simmonds & McDonald, 1984) the percentage successful culture, at 69% overall, is the same. Kaiser *et al.* (1981) had 80% successful culture of lung tumours using a similar agar system, but only

37% of the tumours were lung primaries. Similarly, Bertelsen *et al.* (1983) claimed 79% successful culture but here also, nearly 50% of the tumours studied were metastatic. A higher growth rate is to be expected with metastatic tumours as the process of metastasis has already selected those subpopulations with a greater potential for growth. All tumours received by us were of primary lung origin and if this type of tumour is compared, then growth rates are 72% (Bertelsen *et al.*, 1983) and 75% (Kaiser *et al.*, 1981).

Our yield of drug results was 23% for medium 1, 93% for medium 4 and a potential for 60% and 57% in media 5 and 6 respectively. Kaiser *et al.* had 56% drug results for primary tumours and Bertelsen *et al.* had 72% for a similar group. What is not clear about their results, however, is whether plating efficiencies were significant. Both groups accepted minimum PE of only 0.006 or 0.008 which is exceedingly low and it is not stated to what extent these minimum figures were exceeded.

Analysis of drug results shows that for medium 1, where area under the curve was used as an index of sensitivity, 61% of samples were sensitive to vindesine, and only 15% were sensitive to *cis*-platinum. Thirty-nine percent of samples were clearly resistant to vindesine, while 69% were resistant to platinum exluding the 16% who showed intermediate response. When REL medium was used for drug study and evaluation of sensitivity done on a percentage survival basis, 45% of samples were sensitive to platinum and 55% sensitive to vindesine. Similar pictures emerged from both studies, that of either pronounced sensitivity to drugs or of overt resistance and a superior response rate to vindesine over platinum *in vitro*. When individual patients are evaluated for their sensitivity to both drugs, 36% were sensitive using REL (medium 4) compared with 17% in medium 1. Thirty-six per cent of patients were resistant to both drugs with REL compared with 28% for medium 1. These figures of 30–40% response rates do concur with the clinical experience with these drugs (Elliott *et al.*, 1984). Additionally, although the figures suggested for response in medium 1 for both vindesine and platinum are out of line with clinical experience particularly as *in vivo* vindesine response is poor, the figures suggested for resistance do concur with those expected to be non-responders (60–70%). Again, in this study, no relationship was observed between tumour type and drug sensitivity *in vitro*.

Kaiser *et al.* used 1 h drug exposures to drugs tested with a sensitivity evaluation of $<25\%$ survival at peak plasma concentrations. Results for platinum showed 33% response and 56% of lung tumours tested showed response to at least one drug *in vitro*. Our own observation is that 65% of all samples tested show response to at least one drug *in vitro*. We are satisfied, therefore, that this test bears a relationship to projected clinical response for both platinum and combination performance, and as such, may be used to investigate other drugs. What is not clear, however, is why vindesine consistently gives superior *in vitro* sensitivity results in this assay. This is not the clinical experience, since vindesine as a single agent has not proved itself in the management of NSCCL. It is possible that the concentrations used in the first assay were rather high and resulted in exaggerated figures for sensitivity. This does not explain, however, either the low response rates to platinum in this system or the fact that when the concentrations were changed, vindesine again produced better results (medium 4, REL) although the figures for platinum response were closer to clinical experience.

With respect to growth in various media, the poorer results for the second evaluation of REL against McCoy's rbc require explanation. Blood from August rats was used to prepare red cells, both for REL and for addition at 1% in McCoy's 5A. All blood came from rats of the same age and it is possible that they were past their prime.

It is our intention to continue lung tumour culture using either McCoy's 5A + rbc + hydrocortisone or HITES + 10% FBS with the cell concentrations and incubation times already described. Restrictions of sample size allow only one cell density to be plated and a pilot study established linearity of colony formation over a wide range of cell densities studied. Incubation time, at 12 days, is sufficient to evaluate drug sensitivity, there being no change in colony number between 10 and 21 days, when they begin to fragment. This brings the assay within a time scale of clinical usefulness.

The case for the use of rat rbc as the only factor producing significant increases in plating efficiency and successful culture numbers has not been made. HITES + 10% FBS appears to be a useful alternative. A further option might be to use low (5%) $O_2$ tension during incubation as suggested by Courtenay and Mills (1978). However in a limited number of cultures where this was tried (unpublished) no differences were observed in this system.

With respect to the relevance of testing drugs used only at the present time for inoperable patients, it is clear that any future investigation would be better directed towards factors affecting cytotoxicity of existing drugs and to new drugs themselves. The use of present chemotherapeutic regimes has been validated both *in vivo* and *in vitro* and little remains to be gained by further analysis of these drugs alone.

### References

ALBERTS, D.S., SALMON, S.E., CHEN, H.S.G. & 4 others. (1980*a*). *In vitro* clonogenic assay for predicting response of ovarian cancer to chemotherapy. *Lancet*, **ii**, 340.

ALBERTS, D.S., CHEN, H.S.G, & SALMON, S.E. (1980*b*). *In vitro* drug assay: Pharmacologic considerations. In *Cloning of Human Tumour Stem Cells*, (ed.), p. 197. Alan R. Liss, New York.

ANNUAL REPORT OF THE REGISTRAR GENERAL FOR SCOTLAND (1984). Her Majesty's Stationery Office, Edinburgh.

BERTELSEN, C.A., KERN, D.H., KAISER, L.R., MANN, B.D., HOLMES, E.C. & MORTON, D.L. (1983). Biopsy of thoracic neoplasms for assay of chemosensitivity. *Arch. Surg.*, **118**, 1074.

BRADLEY, T.R., TELFER, P.A. & FRY, P. (1971). The effect of erythrocytes on mouse bone marrow colony development *in vitro*. *Blood*, **38**, 353.

CARNEY, D.N., BUNN, P.A., GAZDAR, A.F., PAGAN, J.A. & MINNA, J.D. (1981). Selective growth in serum free hormone supplemented medium of tumour cells obtained by biopsy from patients with small cell carcinoma of the lung. *Proc. Natl Acad. Sci.*, **78**, 3185.

COURTENAY, V.D. & MILLS, J. (1978). An *in vitro* colony assay for human tumours grown in immune-suppressed mice and treated *in vitro* with cytotoxic agents. *Br. J. Cancer*, **37**, 261.

ELLIOTT, J.A., AHMEDZAI, S., HOLE, D. & 6 others. (1984). Vindesine and *cis*-platinum combination chemotherapy compared with vindesine as a single agent in management of non-small cell cancer of lung: A randomized study. *Eur. J. Cancer Clin. Oncol.*, **20**, 1025.

HAMBURGER, A. & SALMON, S.E. (1977). Primary bioassay of human tumour stem cells. *Science*, **197**, 461.

KAISER, L.R., KERN, D.H., CAMPBELL, M.A., MANN, B.D. & HOLMES, E.C. (1981). *In vitro* assessment of antineoplastic therapy. New indication of thoracotomy? *J. Thorac. Cardiovac. Surg.*, **82**, 538.

SHERIDAN, J.W. & SIMMONS, R.K. (1981). Studies on a human melanoma cell line; effect of cell crowding and nutrient depletion on the biophysical and kinetic characteristics of the cells. *J. Cell Physiol.*, **107**, 85.

SIMMONDS, A.P. & McDONALD, E.C. (1984). Ovarian carcinoma cells in culture: Assessment of drug sensitivity by clonogenic assay. *Br. J. Cancer*, **50**, 317.